# Gross hematuria in an immunocompromised male - A case report

**DOI:** 10.1016/j.ijscr.2025.111300

**Published:** 2025-04-18

**Authors:** Kenneth Cintrón Cartagena, Gustavo Christian Colón, Merary Nazario Pérez, Jaime Matta, Adrian Santana Parachini, Javier Castillo Ortiz

**Affiliations:** a St. Luke's Hospital Urology Residency, 917 Av. Tito Castro, Ponce 00733, Puerto Rico; b Ponce Health Sciences University School of Medicine, Sala Ponce, 388 Calle Luis F, Ponce 00716, Puerto Rico; c Ponce Health Sciences University Department of Surgery, Sala Ponce, 388 Calle Luis F, Ponce 00716, Puerto Rico

**Keywords:** Merkel cell carcinoma, Bladder metastasis, Immunocompromised, Puerto Rican, Hispanic, Case report

## Abstract

**Introduction and importance:**

Merkel cell carcinoma (MCC) is a rare, aggressive neuroendocrine skin cancer with a high propensity for metastasis. While MCC has been reported in various organs, its metastasis to the bladder is exceedingly uncommon. Bladder infiltration in MCC presents a unique diagnostic and therapeutic challenge in Urology. Only few have been described worldwide, and no cases have been reported in Hispanic males. This is the first published case of an immunocompromised Hispanic patient with MCC metastasis to the bladder and to our knowledge, the first case report worldwide to include narrowband imaging (NBI) in the diagnostic algorithm of MCC to the bladder.

**Case presentation:**

We report the case of a 53-year-old immunocompromised Puerto Rican male diagnosed with metastatic MCC. The patient had previously developed MCC with metastasis to the inguinal lymph nodes and received treatment. Later management involved immunotherapy once node persistence was found. Recent investigation revealed a new metastasis to the urinary bladder, presenting with gross hematuria. Diagnostic evaluation included computed tomography (CT) and Cystoscopy with white light and NBI, which confirmed the bladder metastasis. The patient underwent transurethral resection of the bladder tumor (TURBT) for tissue diagnosis. This work has been reported in line with the SCARE criteria (Sohrabi et al., 2023).

**Clinical discussion:**

Histopathological analysis confirmed the diagnosis of metastatic MCC to the urinary bladder. The patient was managed with nephrostomy tube placement for obstructive uropathy and further immunotherapy for metastatic disease. Despite intervention, the prognosis remains poor due to the aggressive nature of the tumor and the patient's immunocompromised state.

**Conclusions:**

This case highlights the unusual presentation of MCC with bladder metastasis in an immunocompromised Hispanic male. The patient revealed aggressive pathology per CT and developed persistent hematuria which were managed with bilateral nephrostomy for symptom relief. To our knowledge, this is the first reported case of MCC bladder metastasis in a Puerto Rican immunocompromised male, underscoring the importance of considering rare metastatic sites in MCC and tailoring treatment approaches accordingly.

## Highlights

• Merkel cell carcinoma is of neuroendocrine origin.
• This malignancy is associated with suppression of the immune system.
• Metastasis of this Merkel cell carcinoma to the urinary bladder is rarely documented in medical literature.
• This is the first recorded case of a Puerto Rican with this metastasis.
• This is the first time that narrow band imaging technique is used for identifying this malignancy.

## 1 Introduction

Merkel cell carcinoma (MCC) is a rare and aggressive skin cancer originating from cutaneous neuroendocrine Merkel cells due to sun exposure or immune-mediated pathways [1]. It has a low incidence, with about 2500 new cases diagnosed annually in the U.S. [1]. MCC has a poor prognosis, particularly in metastatic stages, due to its rapid growth and high metastatic potential [2]. The condition primarily affects Caucasians over 50 years of age with a history of significant sun exposure, chronic immune suppression, or Merkel cell polyomavirus (MCPyV) infection [2]. Clinically, MCC presents as a firm, erythematous nodule on sun-exposed skin [3]. The antibody titer to the MCPyV oncoprotein serves as a biomarker for treatment response in some patients [4]. MCC is often misdiagnosed as other malignancies, such as basal cell carcinoma, squamous cell carcinoma, malignant melanoma, lymphoma, or small cell carcinoma, highlighting the need for accurate immunohistochemical analysis [5]. Key markers like cytokeratin 20 (CK20) and neuron-specific enolase distinguish MCC from other skin cancers [6].

MCC commonly metastasizes to fascia, muscle, and bone, but genitourinary tract metastasis, particularly to the bladder, is exceedingly rare, documented only four times [7]. Treatment typically includes surgery, radiation therapy, and chemotherapy, with immunotherapy emerging as a promising option for metastatic cases [8]. Checkpoint inhibitors targeting the PD-1/PD-L1 pathway have improved survival rates, but prognosis remains poor, with a five-year survival rate of 18 % for metastatic MCC [9]. Early detection and a multidisciplinary approach are crucial to improving outcomes. The American Joint Committee on Cancer (AJCC) classifies MCC staging using the TNM system, where advanced stages correlate with decreased survival rates [10]. The National Cancer Data Base reports five-year survival rates ranging from 80 % (Stage IA) to 20 % (Stage IV) [11]. Limited literature exists on MCC metastasizing to the bladder [[12], [13], [14], [15]], highlighting the need for clinical awareness when evaluating bladder tumors, particularly in immunocompromised patients. This case report presents a rare instance of MCC metastasizing to the bladder, emphasizing its clinical presentation, diagnostic challenges, and treatment.

## 2 Case report

A 53-year-old Hispanic/Latino Puerto Rican male presented with left-sided flank pain, dysuria, urinary dribbling, frequency, and hematuria. His medical history included hypertension, diabetes mellitus, chronic kidney disease (CKD), anal cancer (HPV-related), MCC, and HIV infection. Surgical history included right-sided Mediport catheter placement, right knee MCC excision, inguinal lymph node excision, and open surgery for stone removal. Diagnosed with HIV in 1991, he adhered to Darunavir/cobicistat and Dolutegravir therapy, with a CD4 count between 200 and 300 cells/mm^3^.

**Fig. 1:**
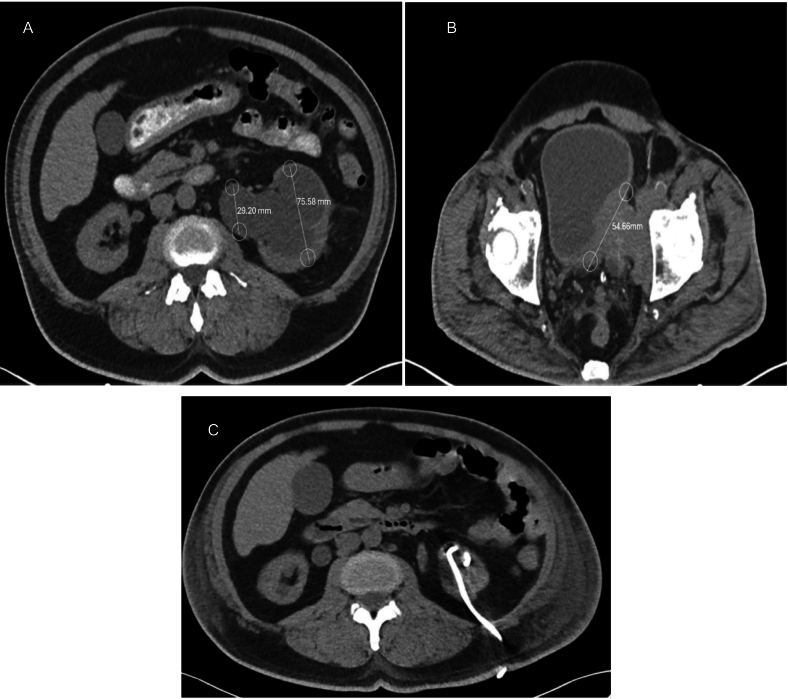
(A–C) NOV 10 2024.

During the COVID-19 pandemic, the patient developed a red papule above the knee, which enlarged significantly before being biopsied and diagnosed as MCC. Subsequent imaging revealed metastasis to the right inguinal lymph nodes. He underwent chemotherapy with etoposide/cisplatin and radiation therapy (RT) in December 2020, followed by pembrolizumab from December 2022 to November 2023. Due to disease progression, avelumab was initiated in December 2023. A recent CT scan indicated left hydronephrosis caused by a 5.47 cm obstructing bladder mass extending into the left lower pelvis, shown in Fig. 1, with associated soft tissue thickening in the right abductor muscle encased the right common femoral artery and vein.

**Fig. 2:**
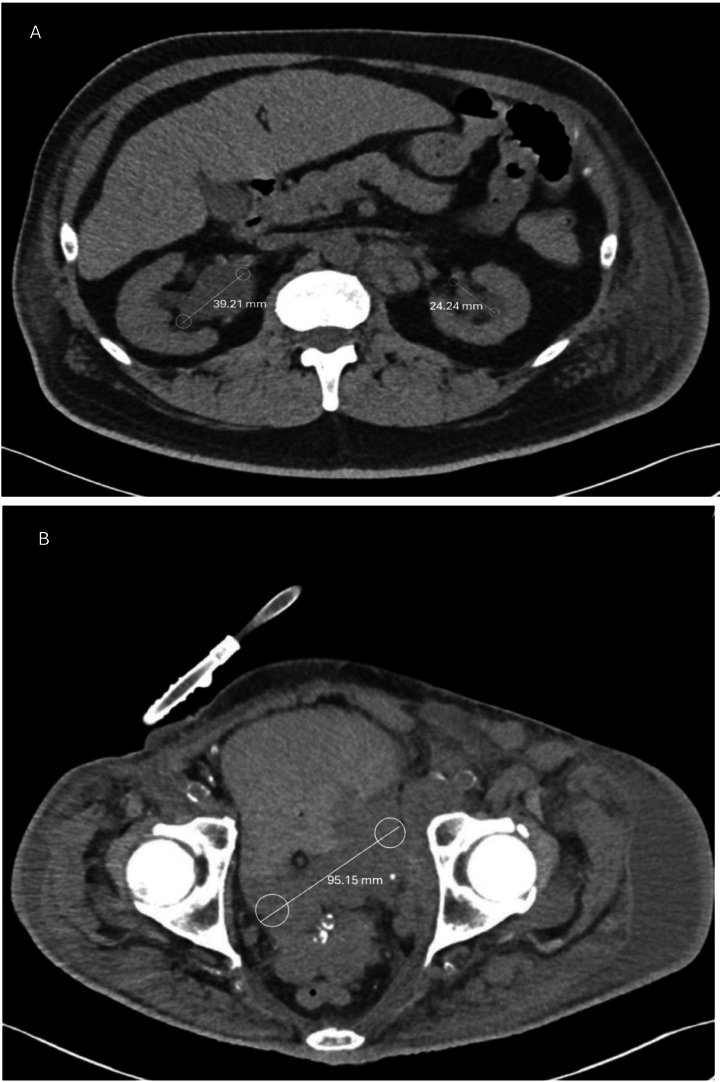
(A–B) (1).

A percutaneous nephrostomy tube (PCNT), shown in Fig. 1, was placed due to complete ureteral obstruction, leading to renal decompression. Three weeks later, the patient was readmitted with gross hematuria, urinary dribbling, and right-sided flank pain. A CT scan showed unchanged bladder mass size but increasing right-sided hydronephrosis. Subsequent imaging revealed significant tumor growth to 9.51 cm, shown in Fig. 2. Laboratory tests showed normochromic normocytic anemia, hyperkalemia, and worsening CKD, with creatinine rising from 4.5 to 5.5 mg/dL over two weeks.

**Fig. 3:**
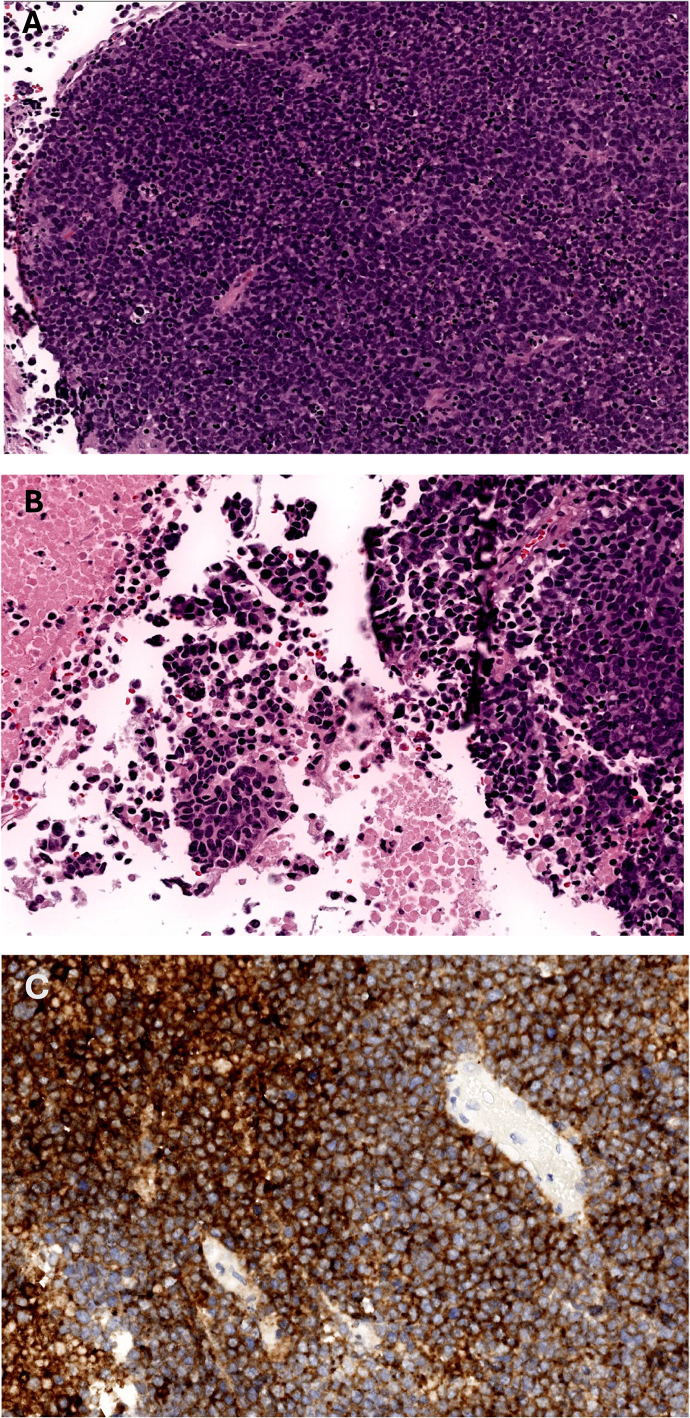

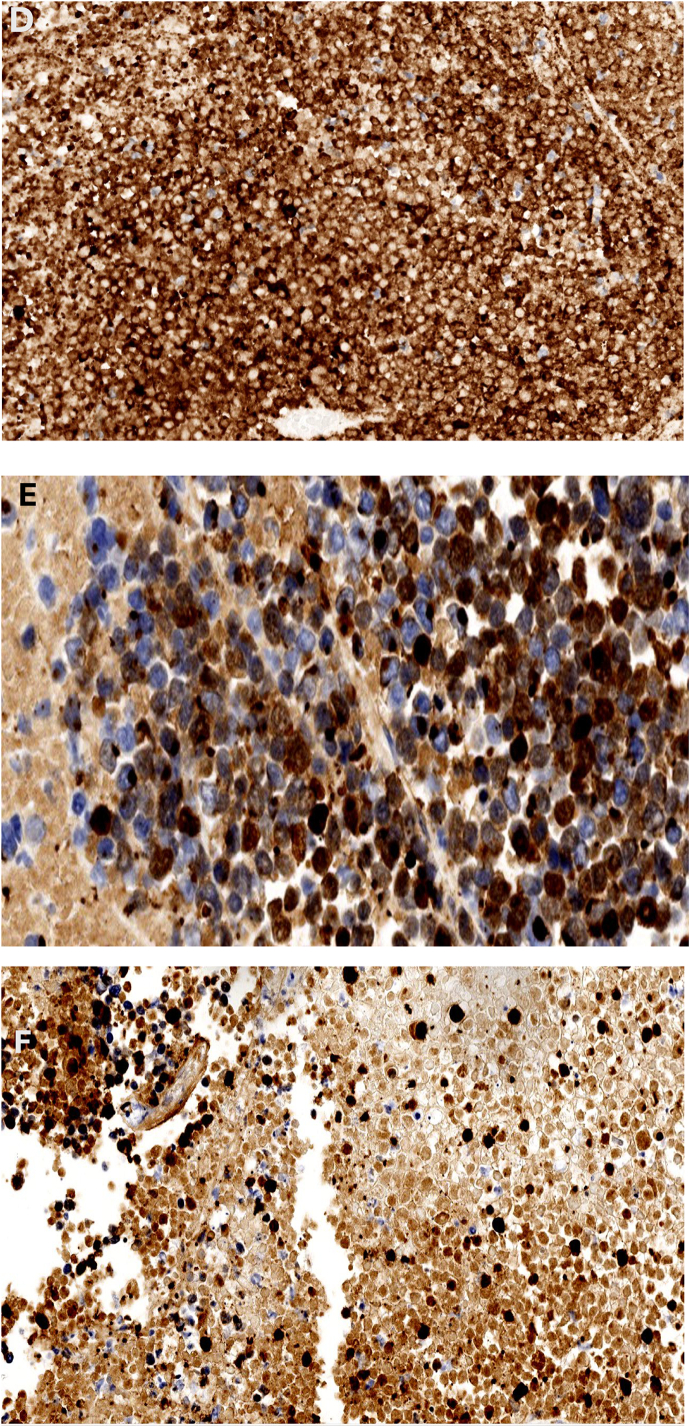
(A–F) November 10.

A three-way Foley catheter with manual constant irrigation was placed for clot evacuation and urinary retention relief. Transurethral bladder resection of the tumor (TURBT) was performed using general anesthesia. Gross examination revealed a 9.5 × 9.0 × 1.0 cm dark red soft tissue mass with necrotic debris. Microscopic analysis identified small round basophilic cells with a high nucleus-to-cytoplasm ratio, dispersed chromatin, and decreased cytoplasm, consistent with neuroendocrine tumors, shown all in Fig. 3. Immunohistochemical staining confirmed MCC, with tumor markers positive for Chromogranin, CD56, Synaptophysin, Cytokeratin AE1/AE3, and CK20 [15]. CD 56 shown in Fig. 3C, Synaptophysin shown in Fig. 3D, Cytokeratin AE1/AE3 shown in Fig. 3E CK20 positivity confirmed its Merkel cell origin, shown in Fig. 3F. Pathology report in Table 1.

**Table 1:**
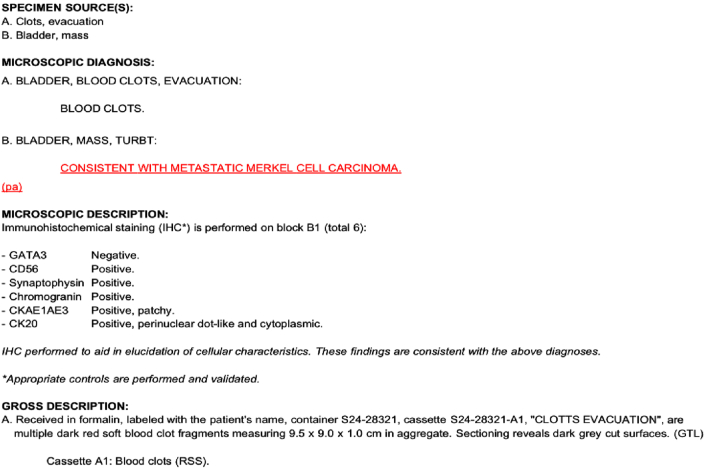
Pathologic Result from Bladder Tumor Resection.

## 3 Discussion

**Fig. 4:**
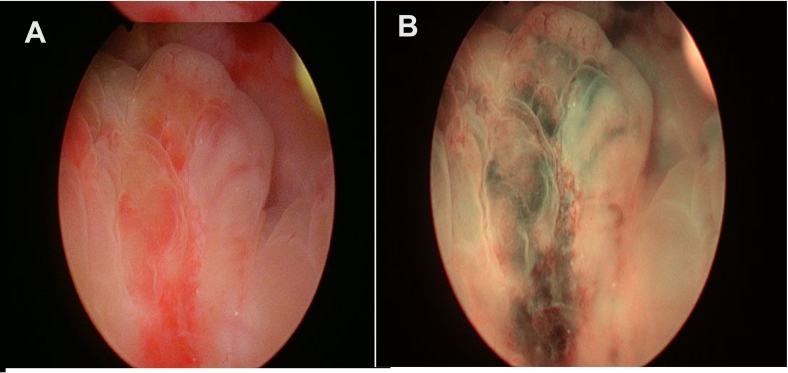
(A–B) November 10.

This case represents an exceptionally rare instance of MCC metastasizing to the bladder. Literature review suggests this is the first case diagnosed via narrow-band imaging (NBI). NBI contributed to diagnosis via improved visualization of vascular structures, which allowed for more efficient resection. NBI also aided in differentiation from other bladder lesions based on hypervascularity and mucosal changes in bladder epithelium. NBI findings are shown in Fig. 4, showing findings before (4A) and after (4B) visualization. Histopathology confirmed the diagnosis, with MCC displaying small, round cells, mitotic figures, and necrosis, consistent with aggressive neuroendocrine carcinoma [16]. Immunohistochemical markers confirmed MCC's epithelial and neuroendocrine origin, with CK20 further verifying its Merkel cell lineage [17].

Previous case reports describe chemotherapy regimens using vinblastine and cisplatin or carboplatin and etoposide for palliative care [[12], [13], [14]]. Current guidelines from the National Comprehensive Cancer Network (NCCN) recommend systemic therapy and RT for distant MCC metastases [18]. Immunotherapy, particularly checkpoint inhibitors like pembrolizumab, has demonstrated durable responses [19]. The combination of platinum-based chemotherapy and topoisomerase inhibitors, such as cisplatin/etoposide, remains a standard approach [18]. Surgery may be beneficial for symptom control in metastatic MCC [18].

This patient's treatment aligned with NCCN guidelines for metastatic MCC. Checkpoint inhibitors (CPIs) are now frontline therapies for advanced MCC, showing promising results [19]. Staging using the TNM system classified this case as Stage IV, correlating with a poor prognosis [18]. Immunosuppression due to HIV infection likely contributed to MCC progression, as HIV-positive individuals have a 13-fold increased risk of MCC compared to the general population [20]. This association underscores the aggressive nature of MCC in immunocompromised individuals and the importance of early detection and tailored therapy.

## 4 Conclusion

This case highlights the rarity of MCC metastasizing to the bladder and underscores the importance of a multimodal management approach. Clinicians should consider MCC in the differential diagnosis of bladder masses, particularly in immunocompromised patients. Further research is needed to improve treatment strategies for distant MCC metastases. Vigilance and early intervention remain crucial for improving patient outcomes.

## CRediT authorship contribution statement

Kenneth Cintrón Cartagena: Study concept, writing the paper, data collection, data analysis and interpretation

Gustavo Christian Colón: Writing the paper, data collection, data analysis

Merary Nazario Pérez: Writing the paper, data collection, data analysis

Jaime Matta: Writing the paper, data collection

Adrian Santana Parachini: Study concept, interpretation of data

Javier Castillo Ortiz: Writing the paper, study concept, data analysis, data interpretation

## Consent

For this case report, informed consent was performed and patient signed consent form to carry out this case report. Evidence is available for review by editor upon request.

## Ethical approval

Approved by IRB at local institution. **IRB Protocol Number:** 2408212354.

## Sources of funding

No funding used to write this case report.

## Guarantors

Kenneth Cintron Cartagena

Gustavo Christian Colon

## Registration of research studies

This case report is not a “first in man” study.

## Declaration of competing interest

No conflict of interests to report.
